# Cholesterol metabolism—physiological regulation and pathophysiological deregulation by the endoplasmic reticulum

**DOI:** 10.1007/s10354-018-0626-2

**Published:** 2018-02-27

**Authors:** Clemens Röhrl, Herbert Stangl

**Affiliations:** Department of Medical Chemistry, Center for Pathobiochemistry and Genetics, Medical University of Vienna, Währingerstraße 10, 1090 Vienna, Austria

**Keywords:** Transcription factors, Atherosclerosis, Unfolded protein response, Endoplasmic reticulum stress, Transkriptionsfaktoren, Atherosklerose, „Unfolded protein response“, Stress im endoplasmatischen Retikulum

## Abstract

Cholesterol is an essential lipid for mammalian cells and its homeostasis is tightly regulated. Disturbance of cellular cholesterol homeostasis is linked to atherosclerosis and cardiovascular diseases. A central role in the sensing and regulation of cholesterol homeostasis is attributed to the endoplasmic reticulum (ER). This organelle harbours inactive transcription factors, which sense ER cholesterol levels and initiate transcriptional responses after activation and translocation into the nucleus. Thereupon, these responses enable adaption to high or low cellular cholesterol levels. Besides the abovementioned canonical functions, ER stress—induced by metabolic burden—and the resulting unfolded protein response influence cholesterol metabolism relevant to metabolic disorders. This review summarizes basic as well as recent knowledge on the role of the ER in terms of regulation of cholesterol metabolism.

## Zusammenfassung

Cholesterin ist ein integraler Bestandteil menschlicher Zellen, daher wird seine Homöostase engmaschig reguliert. Ein Ungleichgewicht im Cholesterinhaushalt führt zu Atherosklerose und in der Folge zu kardiovaskulären Erkrankungen. Die zentrale Schaltstelle der Zelle im Cholesterinhaushalt ist das endoplasmatische Retikulum (ER), welches seine Cholesterinkonzentration in einem sehr engen Rahmen mittels Transkriptionsfaktoren konstant hält. Dazu wird über inaktive Transkriptionsfaktoren der Cholesterinspiegel erfasst, und nach Aktivierung und Translokation in den Zellkern werden Transkriptionsreaktionen ausgelöst. Im Laufe der Jahre wird die metabolische Leistungsfähigkeit des ER beeinträchtigt, v. a. durch das Überangebot an Nahrung. Diese wie auch andere metabolische Ereignisse induzieren den sog. ER-Stress, ein Adaptationsmechanismus der Zelle auf kurzfristige Überbelastung. Dieser wird durch Proteinkaskaden, die Reaktion auf ungefaltete Proteine, „unfolded protein response“ (UPR) ausgelöst. Eine chronische Überlastung des ER führt auch zu einer Veränderung im Cholesterinstoffwechsel. Die vorliegende Übersichtsarbeit stellt eine Zusammenfassung des aktuellen Wissens über die Rolle des ER im Cholesterinhaushalt sowie dessen Beeinflussung durch chronische metabolische Belastung dar.

## Cholesterol function and its role in atherosclerosis

Clinically, the most important plasma lipids are triglycerides and cholesterol. Apart from the central role of cholesterol in cellular organization and stability, it serves as a building block for steroid hormones, vitamin D, oxysterols and bile acids [1]. Its insolubility in plasma requires its transport in spherical macromolecules called lipoproteins, which consist of a hydrophobic core, containing mainly cholesteryl esters and triglycerides, and a hydrophilic coat consisting of phospholipids, free cholesterol and apolipoproteins. The major cholesterol-carrying lipoproteins are low-density lipoprotein (LDL; Table 1) and high-density lipoprotein (HDL) [1]. The clinical relevance of plasma lipid and lipoprotein levels for diagnosis and prognosis of diseases like atherosclerosis is generally accepted. An imbalance in cholesterol homeostasis is triggered by increased food intake or by genetic factors resulting in disposal of cholesterol in peripheral tissues, for instance the arterial wall. This can lead to atherosclerosis, vessel dysfunction and blocked blood flow, the underlying mechanism for cardiovascular diseases (CVDs). CVDs include stroke, heart attack and peripheral arterial disease (PAD) among others, and are still the number one killer in the westernized world [1].

**Table 1:** Selected factors implicated in cholesterol metabolism

Factor	Full name	Function
ABCA1	ATP binding cassette subfamily A member 1	Cholesterol export protein; key factor in HDL biogenesis
ABCG1	ATP binding cassette subfamily G member 1	Cholesterol export protein
ABCG5	ATP binding cassette subfamily G member 5	Biliary sterol exporter
ABCG8	ATP binding cassette subfamily G member 8	Biliary sterol exporter
Apo	Apolipoprotein	Structural component of lipoproteins; stabilizes lipoproteins and is an interaction partner for cellular receptors
ATF4	Activating transcription factor 4	Downstream signalling protein of the UPR; activated by PERK
ATF6	Activating transcription factor 6	Downstream signalling protein of the UPR; mediates transcription of chaperones
GRP78	Glucose-related protein 78	ER chaperone
HDL	High-density lipoprotein	Cholesterol transport vehicle; main factor in RCT; transports cholesterol to the liver and steroidogenic tissues
HMG-CoAR	3-hydroxy-3-methylglutaryl-CoA reductase	Rate-liming enzyme in cholesterol biosynthesis; target of statins
IDOL	Inducible degrader of the LDLR	Negative regulator of LDL uptake
INSIG	Insulin-induced gene	Blocks SREBP activation in response to high cellular cholesterol levels
IRE1	Inositol-requiring enzyme 1	Downstream signalling protein of the UPR; highly conserved among species
LDL	Low-density lipoprotein	Main cholesterol transport vehicle in humans
LDLR	Low-density lipoprotein receptor	Main receptor supplying cells with cholesterol
LXR	Liver X receptor	Positive regulator of cholesterol efflux and fatty acid synthesis
NRF1	Nuclear respiratory factor 1; official gene name: NFE2L1 (nuclear factor, erythroid 2 like 1)	Transcription factor which is activated in response to high cellular cholesterol levels; positive regulator of cholesterol efflux
PCSK9	Proprotein convertase subtilisin-like kexin type 9	Negative regulator of plasma LDL levels; mediates degradation of the LDLR
PERK	Protein kinase RNA-like ER kinase	Downstream signalling protein of the UPR; excess PERK-activation triggers apoptosis
RCT	Reverse cholesterol transport	Transport of excess peripheral cholesterol into the liver for disposal into the bile
S1P, S2P	Site- 1/2 protease	Golgi-resident proteases which activate transcription factors involved in cholesterol homeostasis (SREBP) and unfolded protein response (ATF6)
SCAP	SREBP cleavage-activating protein	Mediates transport of SREBP from the ER to the Golgi apparatus
SR-BI	Scavenger receptor class B member 1	HDL receptor
SREBP	Sterol regulatory element binding protein	Transcription factor which is activated in response to low cellular cholesterol levels; positive regulator of cholesterol uptake and synthesis
UPR	Unfolded protein response	Signalling cascade activated by ER stress; mediates adaptive mechanisms or apoptosis

Atherosclerosis is initiated by endothelium damage leading to the migration of monocytes into the intima and differentiation into macrophages. Macrophage scavenger receptors mediate excessive endocytosis of modified LDL, which lack negative feedback regulation and result in macrophage foam cell formation. The excessive uptake of modified LDL leads to cholesterol-induced apoptosis and inflammation. Cytokines produced by macrophages and endothelial cells stimulate smooth muscle cell proliferation. These early lesions are called fatty streaks and are precursors for advanced atherosclerotic lesions. The latter are characterized by a fibrous cap consisting of smooth muscle cells and extracellular matrix enclosing the lipid-enriched necrotic core. Non-resolved inflammation finally leads to plaque expansion, destabilization and rupture [2].

Taken together, cholesterol is an essential membrane constituent of mammalian cells; however, excess intracellular cholesterol can be toxic and leads to foam cell formation and cell stiffening, which in turn affects vascular integrity and cell signalling. Therefore, cells need to keep a tight balance between cholesterol synthesis, uptake and export, as cholesterol itself cannot be degraded in higher organisms. For cellular cholesterol efflux, cholesterol is transferred to HDL particles, which accept excess cholesterol mainly from peripheral cells and tissues for its transport back to the liver for concomitant disposal into the bile. This pathway is called reverse cholesterol transport (RCT). In contrast, circulating LDL particles, which are formed from triglyceride-rich lipoproteins after remodelling in the plasma and the liver, accomplish cholesterol transport to cells requiring lipids. In this case, cells express higher levels of the LDL-receptor (LDLR), a protein that mediates uptake of LDL particles via the classical receptor-mediated endocytosis pathway [3]. In case of total body cholesterol overloading, cholesterol will ultimately accumulate in LDL particles, which results in their prolonged circulation in the blood stream. This imbalance is consequently followed by oxidation of the particle itself, leading to increased atherosclerotic potential and stiffness of cell membranes. Indeed, oxidized LDL particles have been shown to induce endothelial stiffness [4]. The atherogenic process might, in turn, alter other transport processes, like HDL transcytosis over endothelial cells, which is crucial for lipid clearance in the intima. Over time, cholesterol and other lipids are deposited at the vessel wall, leading to plaque formation. However, the whole phenomenon starts at the cellular level when cells are confronted with cholesterol overload. Thus, understanding of the intracellular stress reactions triggered by cholesterol overload is necessary in order to undertake pharmacological counteractions.

## Cellular cholesterol distribution and trafficking

Cholesterol is heterogeneously distributed throughout cellular membranes and organelles. It is enriched in the plasma membrane (PM), whereas endoplasmic reticulum (ER) cholesterol content is low. Specifically, cholesterol is 5–10-fold enriched in the PM compared to the ER. Cholesterol is also abundant in the endosomal recycling compartment (ERC), multivesicular bodies (MVBs), the trans-Golgi network and the Golgi apparatus [5]. Its heterogeneous distribution throughout cells is maintained to generate membrane properties compatible with the organelles’ distinct functions: Higher cholesterol concentrations contribute to increased membrane thickness and reduced permeability to small molecules, consistent with the barrier function of the plasma membrane. In contrast, low cholesterol concentrations in the ER are associated with increased membrane flexibility, enabling insertion and folding of proteins in its lipid bilayer [6].

**Fig. 1:**
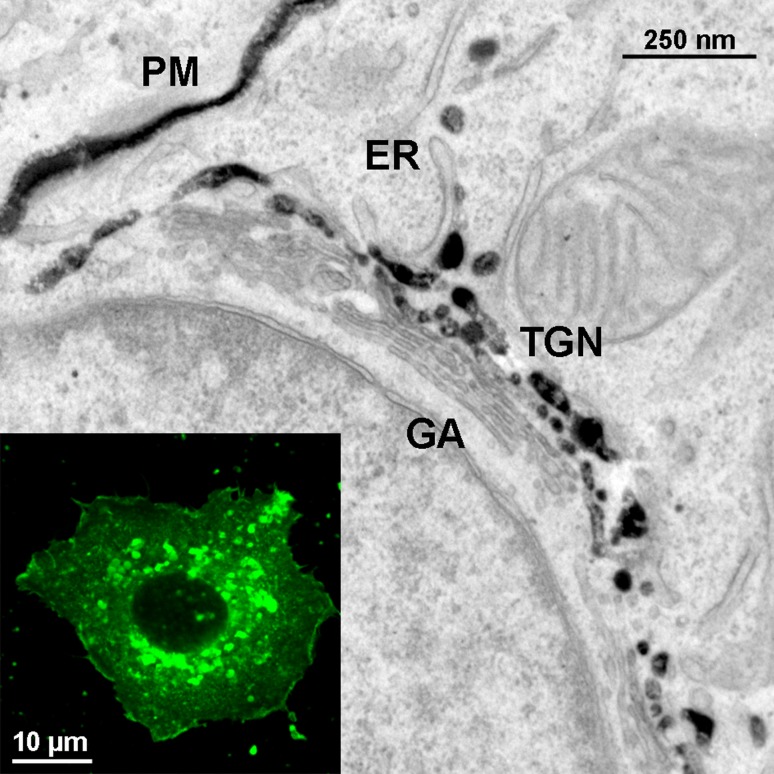
Investigation of cholesterol localization at high resolution. The inset shows a representative hepatic HepG2 cell after incubation with high-density lipoprotein (HDL) labelled with the fluorescent cholesterol analogue Bodipy-cholesterol (Bodipy: boron dipyrromethene difluoride; coloured in *green*). Cholesterol is found at the cell membrane and is enriched in the perinuclear area. The full picture represents a hepatic cell equally incubated with HDL containing Bodipy-cholesterol after diaminobenzidine photooxidation, which in turn leads to formation of electron-dense precipitate, enabling the visualization of fluorescent cholesterol itself via electron microscopy. Cholesterol localization is indicated by dark, electron-dense staining at the plasma membrane (*PM*), the trans-Golgi network (*TGN*) and in the trans-most cisterna of the Golgi apparatus (*GA*), whereas the endoplasmic reticulum (*ER*) is devoid of staining

Analysis of cholesterol’s subcellular localization had been hindered by the lack of methods to detect cholesterol in cells at high resolution. To overcome this obstacle and to visualize cellular cholesterol distribution by transmission electron microscopy at nanometre resolution, we—in collaboration with Adi Ellinger’s and Margit Pavelka’s group—made use of the development of novel fluorescent reporter probes mimicking cholesterol [7] in combination with methodological advances in photooxidation technology [8]. Using this method we were able to confirm and extend previous biochemical studies on the subcellular localization of cholesterol (Fig. 1; [9]).

The classical LDL-receptor pathway, which is well characterized by the seminal work of Brown and Goldstein, accounts for the majority of exogenous cholesterol delivery to most cell types [3]. In addition, HDL is an important source of cholesterol for specialized cell types such as cells involved in steroidogenesis [10].

LDL-receptor-bound LDL is internalized via clathrin-coated pits, which fuse with early sorting endosomes. Here, the LDL-receptor dissociates from its ligand as a result of decreased pH and then recycles back to the PM. Importantly, LDL-receptor recycling is inhibited by proprotein convertase subtilisin-like kexin type 9 (PCSK9), which is the target of the latest generation of cholesterol-lowering drugs [11]. The majority of LDL particles are further delivered to late endosomes followed by degradation of the particle and export of cholesterol. Intracellularly, cholesterol is transported via vesicular trafficking as well as non-vesicular carrier proteins to the trans-Golgi network, the PM and the ER [12]. In the ER, excess cellular cholesterol is esterified and stored in lipid droplets. These cholesteryl-ester stores can be activated in case of low cholesterol content.

In contrast to the cellular uptake of LDL, the delivery of HDL-derived cholesterol to cells is more complex and versatile. Selective uptake of cholesterol from HDL by scavenger receptor class B, type I (SR-BI) without catabolism of the lipoprotein particle is the major route of cholesterol delivery from HDL to cells [10]. In addition, HDL incorporates into lipid bilayers for direct cholesterol exchange [13]. Finally, endocytosis of HDL followed by lipid exchange and resecretion of the lipoprotein particle contributes to cellular cholesterol homeostasis [14, 15].

## Canonical regulation of cholesterol homeostasis in the endoplasmic reticulum

Cellular cholesterol homeostasis is tightly regulated by cholesterol synthesis, uptake from lipoprotein particles and efflux to extracellular acceptors. Cholesterol can be synthetized in the ER from acetate [16]. The rate-limiting enzyme is hydroxymethylglutaryl-CoA reductase (HMG-CoAR), which is tightly regulated and catalyses an irreversible step, the synthesis of mevalonate. Mevalonate is further converted to squalene and then to lanosterol and finally, in mammals, to cholesterol. Newly synthesized cholesterol leaves the ER towards the plasma membrane either by vesicular transport along the secretory pathway or by non-vesicular transport, with the latter being the preferred mechanism [17, 18]. Importantly, HMG-CoAR is competitively inhibited by statins, the classical cholesterol-lowering drugs. Inhibition of cholesterol synthesis by statins leads to compensatory up-regulation of LDL-cholesterol import and consequently to lower plasma cholesterol levels.

Although the cholesterol content of the ER is very low, it is the main organelle for cholesterol level sensing and regulation. The transcription factor family sterol regulatory element binding proteins (SREBPs) are essentially involved in this regulation [19]. SREBPs in the ER are associated with Insulin-induced gene 1 protein (INSIG) and the SREBP cleavage activating protein (SCAP), which has a sterol-sensing domain. Decreasing cholesterol levels (below 5%) in the ER membrane lead to a conformational change of SCAP, which results in dissociation of INSIG1 from the complex [20]. The SREBP-SCAP complex can now leave the ER and is transported to the Golgi apparatus, where the proteases S1P and S2P release active SREBP, which translocates to the nucleus. Especially SREBP-2 controls cholesterol pathways [21], and target genes include HMG-CoAR, LDL-receptor and the HDL receptor SR-BI. Thus, decreasing ER cholesterol levels result in increased cholesterol synthesis and uptake regulated via SREBP2. Besides its physiological role as a sensor and modulator of cellular cholesterol metabolism, SREBP2 is implicated to play a pathophysiologically relevant role in a variety of disorders such as insulin resistance, pancreatic β‑cell toxicity, cognitive dysfunction and cancer [22]. For instance, SREBP2 is activated by low extracellular pH by an unknown mechanism. This might provide a growth advantage to cancer cells in the acidified tumour microenvironment [23]. Finally, SREBP2 is activated by ER stress, independent of cellular cholesterol level [24], as described in detail below (paragraph 4).

While SREBP2 prevent cells from insufficient cholesterol supply, a recent discovery identified a novel mechanism that prevents the ER from accumulation of excess cellular cholesterol [25]: the ER-bound transcription factor nuclear factor erythroid 2 related factor-1, Nfe2L1, often called NRF1. NRF1 directly binds cholesterol and thereby senses high cholesterol levels in the ER, leading to a de-repression of genes involved in cholesterol removal. This activation of cholesterol excretion is mediated by the liver X receptor (LXR). On the contrary, NRF1 deficiency leads to massive cholesterol accumulation in the liver. Similarly, oxysterols, which are formed in amounts proportional to cholesterol, regulate cholesterol homeostasis. Oxysterols are also ligands of NRF1, thereby mediating an LXR response. LXR itself forms heterodimers with the retinoid X receptor after activation and stimulates the expression of target genes [26]. Important target genes are the ATP binding cassette transporters (ABC) ABCA1 and ABCG1, which increase cholesterol efflux, and the inducible degrader of the LDL-receptor (IDOL), which down-regulates the LDL-receptor pathway [27]. Furthermore, the LXR pathway is a promising target for pharmacological activation to attenuate atherosclerotic plaque development [26], which is still limited because of adverse effects of available LXR agonists.

## Deregulation of cholesterol homeostasis by endoplasmic reticulum stress

ER functions are not restricted to protein synthesis. Instead, the ER can be envisioned as a central sensor receiving systemic input from the whole organism. Especially metabolic syndrome and obesity pose a large burden, as elevated protein synthesis, high plasma levels of insulin and free fatty acid as well as systemic inflammation disturb ER homeostasis, leading to ER stress. Similarly, metabolic deregulation in cancer challenges the ER [28–31]. In non-alcoholic fatty liver disease, a dysbalance of hepatic fatty acids causes ER stress [32]. Of note, also uptake of single meals composed of glucose or fat causes ER stress, indicating that transient loss of ER homeostasis is a physiologically relevant process [33]. However, chronically persistent ER stress—which is the case in metabolic disorders—leads to loss of metabolic control [34].

An elaborate signalling network, referred to as the “unfolded protein response” (UPR) enables adaption to ER stress or—if adaptive mechanisms fail—triggers apoptosis [35]. This signalling network is initiated by three effector proteins, ATF6, IRE1 and PERK, which mediate the activation of a transcriptional program with the aim to increase the ER folding capacity and reduce the protein folding burden of the ER by translational inhibition [35].

Two important discoveries particularly illustrate the interconnection between ER stress and cholesterol metabolism: First, ATF6 and SREBPs are cleaved by the very same proteases in the Golgi apparatus for subsequent translocation to the nucleus [36]. Second, ER stress and apoptosis are central mechanisms in cholesterol-mediated toxicity in macrophage foam cells [37] and therefore negatively affect the development of atherosclerosis. Relevant to atherosclerosis and metabolic disorder, homocysteine elevates cholesterol synthesis via an ER-stress-dependent mechanism [38]. Furthermore, activation of the UPR in hepatic cells decreases expression of ABCA1 [39]. Specifically, the PERK downstream target ATF4 is a regulator of ABCA1 expression [40]. Besides ABCA1, also SR-BI is repressed by the UPR [41]. These cholesterol transporters are involved in formation of HDL and the clearance of HDL-cholesterol from the bloodstream, respectively. This might negatively impact HDL’s reverse cholesterol transport capacity, resulting in peripheral cholesterol accumulation. In obese mice lacking the leptin receptor, over-expression of GRP78 increased biliary cholesterol excretion, indicating that ER dysfunction has an important role in the function of the biliary cholesterol transport proteins ABCG5 and ABCG8 [42]. This mechanism might further contribute to decreased RCT due to lower cholesterol excretion. In contrast, genetic disruption of the IRE1 signalling pathway lowers bile acid synthesis as well as plasma cholesterol levels, which suggests a positive role of the UPR on RCT [43]. Measuring RCT in vivo by monitoring the faecal excretion of cholesterol from cholesterol-loaded macrophages under conditions that interfere with the UPR would provide important insights into the question of how the UPR influences RCT. This would also clarify if chemical chaperones, which assist protein folding and antagonize the UPR in the liver [44], are therapeutically promising agents to counteract atherosclerosis.

## References

[1] Hegele RA (2009). Plasma lipoproteins: genetic influences and clinical implications. Nat Rev Genet.

[2] Glass CK, Witztum JL (2001). Atherosclerosis. the road ahead. Cell.

[3] Goldstein JL, Brown MS, Anderson RG, Russell DW, Schneider WJ (1985). Receptor-mediated endocytosis: concepts emerging from the LDL receptor system. Annu Rev Cell Biol.

[4] Oh MJ, Zhang C, LeMaster E, Adamos C, Berdyshev E, Bogachkov Y (2016). Oxidized-LDL signals through rho-GTPase to induce endothelial cell stiffening and promote capillary formation. J Lipid Res.

[5] Maxfield FR, van Meer G (2010). Cholesterol, the central lipid of mammalian cells. Curr Opin Cell Biol.

[6] Lippincott-Schwartz J, Phair RD (2010). Lipids and cholesterol as regulators of traffic in the endomembrane system. Annu Rev Biophys.

[7] Wustner D, Lund FW, Rohrl C, Stangl H (2016). Potential of BODIPY-cholesterol for analysis of cholesterol transport and diffusion in living cells. Chem Phys Lipids.

[8] Meißlitzer-Ruppitsch C, Röhrl C, Neumüller J, Pavelka M, Ellinger A (2009). Photooxidation technology for correlated light and electron microscopy. J Microsc.

[9] Rohrl C, Meisslitzer-Ruppitsch C, Bittman R, Li Z, Pabst G, Prassl R (2012). Combined light and electron microscopy using diaminobenzidine photooxidation to monitor trafficking of lipids derived from lipoprotein particles. Curr Pharm Biotechnol.

[10] Trigatti BL, Krieger M, Rigotti A (2003). Influence of the HDL receptor SR-BI on lipoprotein metabolism and atherosclerosis. Arterioscler. Thromb. Vasc. Biol..

[11] Horton JD, Cohen JC, Hobbs HH (2007). Molecular biology of PCSK9: its role in LDL metabolism. Trends Biochem Sci.

[12] Ikonen E (2008). Cellular cholesterol trafficking and compartmentalization. Nat. Rev. Mol. Cell. Biol..

[13] Plochberger B, Rohrl C, Preiner J, Rankl C, Brameshuber M, Madl J (2017). HDL particles incorporate into lipid bilayers—a combined AFM and single molecule fluorescence microscopy study. Sci Rep.

[14] Rohrl C, Stangl H (2013). HDL endocytosis and resecretion. Biochim. Biophys. Acta..

[15] Rohrl C, Pagler TA, Strobl W, Ellinger A, Neumuller J, Pavelka M (2010). Characterization of endocytic compartments after holo-high density lipoprotein particle uptake in HepG2 cells. Histochem Cell Biol.

[16] Bloch K (1992). Sterol molecule: structure, biosynthesis, and function. Steroids.

[17] Raychaudhuri S, Im YJ, Hurley JH, Prinz WA (2006). Nonvesicular sterol movement from plasma membrane to ER requires oxysterol-binding protein-related proteins and phosphoinositides. J Cell Biol.

[18] Heino S, Lusa S, Somerharju P, Ehnholm C, Olkkonen VM, Ikonen E (2000). Dissecting the role of the golgi complex and lipid rafts in biosynthetic transport of cholesterol to the cell surface. Proc Natl Acad Sci USA.

[19] Brown MS, Goldstein JL (1997). The SREBP pathway: regulation of cholesterol metabolism by proteolysis of a membrane-bound transcription factor. Cell.

[20] Radhakrishnan A, Goldstein JL, McDonald JG, Brown MS (2008). Switch-like control of SREBP-2 transport triggered by small changes in ER cholesterol: a delicate balance. Cell Metab.

[21] Horton JD, Goldstein JL, Brown MS (2002). SREBPs: activators of the complete program of cholesterol and fatty acid synthesis in the liver. J Clin Invest.

[22] Shimano H, Sato R (2017). SREBP-regulated lipid metabolism: convergent physiology—divergent pathophysiology. Nat Rev Endocrinol.

[23] Kondo A, Yamamoto S, Nakaki R, Shimamura T, Hamakubo T, Sakai J (2017). Extracellular acidic pH activates the sterol regulatory element-binding protein 2 to promote tumor progression. Cell Rep.

[24] Colgan SM, Tang D, Werstuck GH, Austin RC (2007). Endoplasmic reticulum stress causes the activation of sterol regulatory element binding protein-2. Int J Biochem Cell Biol.

[25] Widenmaier SB, Snyder NA, Nguyen TB, Arduini A, Lee GY, Arruda AP (2017). NRF1 is an ER membrane sensor that is central to cholesterol homeostasis. Cell.

[26] Calkin AC, Tontonoz P (2010). Liver x receptor signaling pathways and atherosclerosis. Arterioscler. Thromb. Vasc. Biol..

[27] Zelcer N, Hong C, Boyadjian R, Tontonoz P (2009). LXR regulates cholesterol uptake through Idol-dependent ubiquitination of the LDL receptor. Science.

[28] Hotamisligil GS (2010). Endoplasmic reticulum stress and the inflammatory basis of metabolic disease. Cell.

[29] Eizirik DL, Cardozo AK, Cnop M (2008). The role for endoplasmic reticulum stress in diabetes mellitus. Endocr Rev.

[30] Pagliassotti MJ, Kim PY, Estrada AL, Stewart CM, Gentile CL (2016). Endoplasmic reticulum stress in obesity and obesity-related disorders: an expanded view. Metab Clin Exp.

[31] Eigner K, Filik Y, Mark F, Schutz B, Klambauer G, Moriggl R (2017). The unfolded protein response impacts melanoma progression by enhancing FGF expression and can be antagonized by a chemical chaperone. Sci Rep.

[32] Fuchs CD, Claudel T, Kumari P, Haemmerle G, Pollheimer MJ, Stojakovic T (2012). Absence of adipose triglyceride lipase protects from hepatic endoplasmic reticulum stress in mice. Hepatology.

[33] Boden G, Song W, Duan X, Cheung P, Kresge K, Barrero C (2011). Infusion of glucose and lipids at physiological rates causes acute endoplasmic reticulum stress in rat liver. Obesity (Silver Spring).

[34] Fu S, Watkins SM, Hotamisligil GS (2012). The role of endoplasmic reticulum in hepatic lipid homeostasis and stress signaling. Cell Metab.

[35] Walter P, Ron D (2011). The unfolded protein response: from stress pathway to homeostatic regulation. Science.

[36] Ye J, Rawson RB, Komuro R, Chen X, Dave UP, Prywes R (2000). ER stress induces cleavage of membrane-bound ATF6 by the same proteases that process SREBPs. Mol Cell.

[37] Feng B, Yao PM, Li Y, Devlin CM, Zhang D, Harding HP (2003). The endoplasmic reticulum is the site of cholesterol-induced cytotoxicity in macrophages. Nat Cell Biol.

[38] Werstuck GH, Lentz SR, Dayal S, Hossain GS, Sood SK, Shi YY (2001). Homocysteine-induced endoplasmic reticulum stress causes dysregulation of the cholesterol and triglyceride biosynthetic pathways. J Clin Invest.

[39] Rohrl C, Eigner K, Winter K, Korbelius M, Obrowsky S, Kratky D (2014). Endoplasmic reticulum stress impairs cholesterol efflux and synthesis in hepatic cells. J Lipid Res.

[40] Fusakio ME, Willy JA, Wang Y, Mirek ET, Al Baghdadi RJ, Adams CM (2016). Transcription factor ATF4 directs basal and stress-induced gene expression in the unfolded protein response and cholesterol metabolism in the liver. Mol Biol Cell.

[41] Eberhart T, Eigner K, Filik Y, Fruhwurth S, Stangl H, Rohrl C (2016). The unfolded protein response is a negative regulator of scavenger receptor class B, type I (SR-BI) expression. Biochem Biophys Res Commun.

[42] Wang Y, Su K, Sabeva NS, Ji A, van der Westhuyzen DR, Foufelle F (2015). GRP78 rescues the ABCG5 ABCG8 sterol transporter in db/db mice. Metab Clin Exp.

[43] Liu X, Henkel AS, LeCuyer BE, Hubchak SC, Schipma MJ, Zhang E (2017). Hepatic deletion of X‑box binding protein 1 impairs bile acid metabolism in mice. J Lipid Res.

[44] Ozcan U, Yilmaz E, Ozcan L, Furuhashi M, Vaillancourt E, Smith RO (2006). Chemical chaperones reduce ER stress and restore glucose homeostasis in a mouse model of type 2 diabetes. Science.

